# Achromobacter cluster related to COVID-19 supply chain issues

**DOI:** 10.1017/ash.2024.66

**Published:** 2024-04-29

**Authors:** Mary Ellen Scales, Megan C. Gallagher, Sarah Haessler, Kristy Lindsey, Michele Maryanski, Manju Mathew, Franklin Moore, Karen Hogan, Morgan Gilmore, Stacey Peters, Kristin Smith

**Affiliations:** 1 Division of Healthcare Quality, Baystate Medical Center, Springfield, MA, USA; 2 Independent Infection Prevention Consultant, Windsor, CT, USA; 3 Department of Medicine, University of Massachusetts Chan Medical School—Baystate, Springfield, MA, USA; 4 Division of Infectious Disease, Baystate Medical Center, Springfield, MA, USA; 5 Department of Microbiology, Baystate Health, Holyoke, MA, USA; 6 Department of Pathology, University of Massachusetts Chan Medical School—Baystate, Springfield, MA, USA

## Abstract

Isolation of an unusual organism, *Achromobacter xylosoxidans*, from 2 cardiac surgical patients on the same day prompted an investigation to search for cases and cause. An extensive review demonstrated a pseudo-outbreak related to practices to conserve laboratory saline due to short supply resulting from supply chain shortage from the coronavirus disease 2019 pandemic.

## Background

*Achromobacter xylosoxidans* is an aerobic nonfermenting aquaphilic gram-negative rod (GNR) widely distributed in the environment. It is uncommonly a source of healthcare-associated infections^1^ and often occurs in immunocompromised patients and patients with cystic fibrosis. *A. xylosoxidans* is reported as a contaminant in clinical specimens and solutions, including from reusable tissue dispensers,^2^ furosemide ampules,^3^ dental care unit waterlines,^4^ and disinfectant solutions,^5^ and has led to outbreaks of nosocomial infections^3,6–8^ and pseudo-outbreaks.^2,5^ We investigated 2 cases of *A. xylosoxidans* found on valve culture following cardiac surgery, which is a rare cause of endocarditis.^9,10^ Our objective was to identify the source(s) and prevent further infections.

## Materials and methods

### Description and setting

On 12/8/21, Infection Control at Baystate Medical Center, a teaching hospital with ∼760 beds in Springfield, Massachusetts, was alerted by Infectious Disease about 2 *A. xylosoxidans* positive culture results collected on the same day (12/3/21) from cardiac surgery patients.

### Case 1

A 60-year-old man with peripheral vascular disease and coronary artery disease was admitted for elective 3-vessel coronary artery bypass grafting and carotid endarterectomy. The patient had active tobacco use and social alcohol use, but no injection drug use. Intraoperatively, transesophageal echocardiogram (TEE) found a 1.5 cm mobile mass on the right aortic valve leaflet, which was excised and sent for pathology and microbiology. Gram stain showed no microorganisms, although GNRs grew on culture, identified as *A. xylosoxidans*. He was started on meropenem. Pathology from the valve mass was consistent with a fibroelastoma with no evidence of endocarditis or infection.

### Case 2

A 57-year-old woman with a history of injection drug use, hepatitis C, and recurrent pancreatitis presented to an outside hospital where she was found to have methicillin-sensitive Staphylococcus aureus (MSSA) bacteremia in the setting of recent relapsed injection drug use. She was treated with cefazolin and transferred to our institution. A TEE showed aortic and tricuspid valve vegetations. She underwent aortic and tricuspid bioprosthetic valve replacements on the same day as Case 1. Both valves were sent for culture, and neither showed microorganisms on Gram stain. GNRs grew from both cultures, identified as *A. xylosoxidans*. Cefazolin was changed to meropenem.

The 2 *A. xyylosoxidans* had the same sensitivity pattern (Table 1), raising concern that the 2 cases were linked.

**Table 1.** Organism susceptibilities

Antibiotic tested	Case #1	Case #2
Amikacin	Resistant	Resistant
Cefepime	Resistant	Resistant
Ceftazidime	Resistant	Not tested
Ciprofloxacin	Intermediate	Intermediate
Gentamicin	Resistant	Resistant
Levofloxacin	Susceptible	Susceptible
Meropenem	Susceptible	Susceptible
Piperacillin–tazobactam	Resistant	Resistant
Tetracycline	Susceptible	Susceptible
Tobramycin	Resistant	Resistant
Trimethoprim/sulfamethoxazole	Susceptible	Susceptible

### Retrospective surveillance

Analysis included literature search for the organism, the unique patient population, common sources, and potential cluster causes. Potential sources of contamination for further investigation were identified, including chlorhexidine gluconate (CHG),^6,7^ water,^1^ and specimen handling and transport to lab.^5,8^ The infection prevention team reviewed perioperative documentation and developed a spreadsheet of variables associated with the patients and procedures.

## Results

### Investigation

Both patients underwent cardiac surgery on the same day, in the same operating room (OR), with the same cardiac surgeon. The specimen was collected from the patient’s own native aortic valve on the same day, and neither patient had had prior cardiac surgery. Both patients had received preoperative cleansing with (CHG) 2% impregnated wipes and prepackaged CHG and alcohol skin prep kits as surgical skin preparation prior to surgery. CHG has been implicated in *A. xylosoxidans* clusters,^6,7^ but neither CHG product lot number was under product recall review.

Both patients had epi-aortic echocardiogram procedures performed in the OR with sterilized probes covered with sterile sleeves and single-use sterile packets of ultrasound gel used. The same heater/cooler machine was used for both patients during cardiopulmonary bypass.

Data mining for *A. xylosoxidans* lab results for the previous 12 months revealed 3 additional positive similar isolates obtained on 12/3/2021. There are 3 patients who had bronchoscopy on the same day as the cardiac patients who had valve surgery, and one of those patients had cardiac surgery a few weeks prior. This raised concern for potential associations with the heater-cooler machine and bronchoscopes.

Surgical services leadership and endoscopy staff were queried about bronchoscopy reprocessing and storage. All 3 bronchoscopes were different serial numbers, one of which was disposable. No commonalities were identified in the endoscope or reprocessing review.

Several potential sources of contamination were considered, including equipment or supplies in the OR, endoscopy reprocessing and handling, or specimen management, either during transport or in the microbiology lab.

The common link identified from the microbiology laboratory was that both specimen types listed (bronchiolar lavage and tissue) required a pretreatment step with sterile saline. During the time these specimens were handled, the laboratory was struggling to maintain the supply of individual-use Microscan prompts normally used for pretreatment of tissue cultures and dilution of lower respiratory cultures. The cluster of infections occurred during a 2-week period when prompts were out of stock due to supply chain issues. Individual-use 1 mL saline tubes had been substituted. This saline supply then became unstable as well, and the microbiology lab began obtaining saline from other departments within the facility. On the day that the *A. xylosoxidans* culture results had been identified, the microbiology lab was using saline that was poured into a sterile specimen cup from the neighboring histology lab while awaiting a shipment of the second-line saline product.

**Figure 1.**
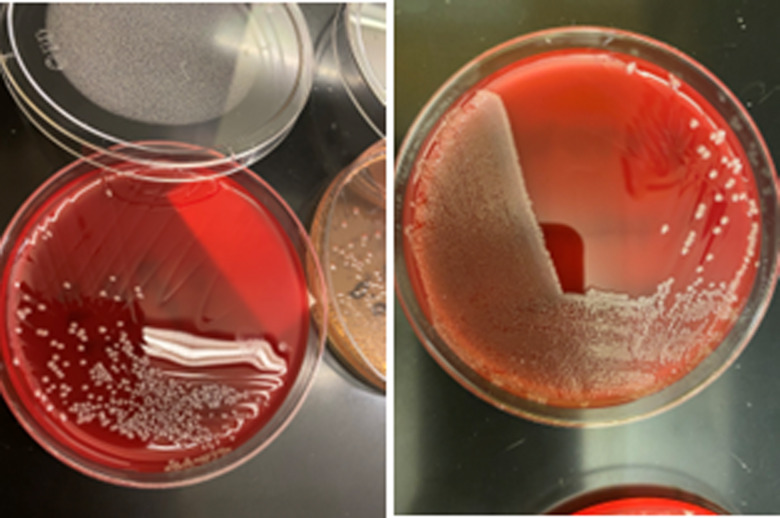
Lab plating *Achromobacter xylosoxidans* from source saline on sheep blood agar.

The borrowed aliquot of saline from the other department was cultured as well as the remaining product in the original opened container. The cultures of the laboratory saline grew *A. xylosoxidans* (Figure 1). These isolates and the isolates from the patients were sent to the Massachusetts Department of Public Health (MDPH) Laboratory, where whole-genome sequencing was performed on 5 clinical specimens and 2 saline specimens used for sample aliquoting in the microbiology lab. All 7 samples appeared clonal with 0 single nucleotide polymorphisms (SNP) differences using a fine-resolution method of whole-genome sequening used in outbreak investigation, Lyve-SET high-quality SNP-based (hqSNP) analysis.

We discovered the original saline container from the neighboring lab department was in use beyond the manufacturer’s recommendations, which are to dispose of the product 60 days after opening. This bottle was originally opened 7/9/21 in the histology laboratory and was still in use when these 5 specimens were processed in December 2021. Additionally, once the saline container was opened, sterility could no longer be guaranteed. The main cause of extended use was the lack of saline product availability due to ongoing supply chain issues of many different products during the coronavirus disease 2019 (COVID-19) pandemic.

At the conclusion, an educational session was developed to share the information with the division of infectious disease, microbiology, and infection control. This emphasized (1) the need for sterile saline for culturing, (2) following expiration dating for preservative-free saline, (3) the expectation that bottle contents are discarded after opening, and (4) that smaller bottles may be more useful in the lab setting. Currently, no further valve cultures have grown *A. xylosoxidans*.

## Conclusions

This case outlines the investigation of a pseudo-outbreak of *A. xylosoxidans* due to contamination of laboratory saline shared among laboratory departments; repackaged and used past manufacturer’s expiration. The extended use and lack of availability were due to supply chain disruptions resulting from the COVID-19 pandemic. Downstream consequences of this pseudo-outbreak included unnecessary broad-spectrum antibiotic exposure for affected patients as well as many hours of time and energy to work up this cluster. This case also emphasizes the need for quality control in specimen handling by laboratory staff to prevent similar errors.

Lessons learned also included the importance of rapid identification of a cluster of unusual organisms and collaboration across multiple disciplines to launch a timely investigation and stop a pseudo-outbreak quickly.
